# Accumulation of Selected Metal Elements in Fruiting Bodies of Oyster Mushroom

**DOI:** 10.3390/foods11010076

**Published:** 2021-12-29

**Authors:** Marcel Golian, Alžbeta Hegedűsová, Ivana Mezeyová, Zuzana Chlebová, Ondrej Hegedűs, Dana Urminská, Alena Vollmannová, Peter Chlebo

**Affiliations:** 1 Institute of Horticulture, Faculty of Horticulture and Landscape Engineering, Slovak University of Agriculture in Nitra, Tr. A. Hlinku 2, 949 76 Nitra, Slovakia; alzbeta.hegedusova@uniag.sk (A.H.); ivana.mezeyova@uniag.sk (I.M.); 2 AgroBioTech Reseach Centre, Slovak University of Agriculture in Nitra, Tr. A. Hlinku 2, 949 76 Nitra, Slovakia; zuzana.chlebova@uniag.sk; 3 Department of Chemistry, Faculty of Education, Selye University, Hradna 21, 945 01 Komarno, Slovakia; hegeduso@ujs.sk; 4 Institute of Biotechnology, Faculty of Biotechnology and Food Sciences, Slovak University of Agriculture in Nitra, Tr. A. Hlinku 2, 949 76 Nitra, Slovakia; dana.urminska@uniag.sk; 5 Institute of Food Sciences, Faculty of Biotechnology and Food Sciences, Slovak University of Agriculture in Nitra, Tr. A. Hlinku 2, 949 76 Nitra, Slovakia; alena.vollmannova@uniag.sk; 6 Institute of Nutrition and Genomics, Faculty of Agrobiology and Food Resources, Slovak University of Agriculture in Nitra, Tr. A. Hlinku 2, 949 76 Nitra, Slovakia; peter.chlebo@uniag.sk

**Keywords:** food safety, fungi, selenium, substrate, *Pleurotus*, heavy metals, mycosorption, mycoremediation

## Abstract

The species *Pleurotus ostreatus* is a commercially, gastronomically, and biotechnologically important fungus. Its strain variability has been little researched. The study provides an evaluation of 59 oyster mushroom production strains in terms of the ability to accumulate selected metals in the cap and stipe. The fruiting bodies were grown under identical model conditions on straw substrate. Metal concentrations (ET-AAS) in dry fruiting bodies ranged in values 1.7–22.4 mg kg^−1^ for Al, 2.6–9.7 mg kg^−1^ Ba, 199–4560 mg kg^−1^ Ca, 1.7–12.0 mg kg^−1^ Cu, 12–120 mg kg^−1^ Fe, 16,000–49,500 mg kg^−1^ K, 876–2400 mg kg^−1^ Mg, 0.39–11.0 mg kg^−1^ Mn, 46–920 mg kg^−1^ Na and 11–920 mg kg^−1^ for Zn. More Cu, Fe, K, Mg, Mn, Zn accumulated in the cap, while in the stipe Ba was amassed. No significant difference was found between Al, Ca and Na between the accumulation in the cap and the stipe. Furthermore, the dependence of metal uptake from the substrate depending on the fortification of the substrate was confirmed. Statistically significant (*p* < 0.05) synergistic relationships were shown in pairs Al and Ba, Al and Fe, Ba and Na, Ba and Ca, Ca and Na, Cu and Fe, Fe and Mn, Fe and Zn, K and Mg, K and Mn, K and Zn, Mg and Mn, Mg and Na, Mg and Zn and Mn and Zn in the substrate without the addition of sodium selenate to the substrate. Altered relationships were observed after the application of sodium selenate to the substrate, synergism of Se and Ni, Se and Co and Se and Hg, Cu and Mn, Cu and Fe, Zn and Co, Zn and Ni, Zn and Hg, Mn and Fe, Mn and Cr, Co and Ni, Co and Hg, Ni and Hg, Pb and Cd. The findings of the study may help in the selection of production strains with hypercumulative properties for a particular metal and subsequent use in the addition of fortified fruiting bodies (e.g., with Zn). Based on the study the strains less sensitive to the accumulation of hazardous metals is possible to select for large-scale production, which is important from the perspective of food safety.

## 1. Introduction

The first attempts to grow the *Pleurotus ostreatus* for human use were carried out during the First World War in Germany. The possibility of producing food using only wood chips, sawdust or straw was an interesting idea. In those years, mushroom cultivation could be an alternative to other foods during the time of the temporary incident. Today, the production of mushrooms on agricultural waste is necessary from the perspective of the large population and the decline of agricultural land. Primally, it eliminates or reduces the amount of environmental waste and secondarily produces food [1]. It is an optimal strategy for the disposal of lignocellulosic materials. In Bangladesh and around the world today, oyster mushroom (*Pleurotus ostreatus*) grown mainly for its great adaptability, medicinal effects [2] and applicability in the diet. *Pleurotus ostreatus* is a mushroom of delicious taste. Glucans, which together with chitin and other hemicelluloses form their cell wall, play a key role in the pharmaceutical use of mushrooms. From the point of view of human medicine, it helps to regulate the body’s immune response, regulates the level of cholesterol and glucose in the blood, affects the activity of macrophages and others [3]. *P. ostreatus* species is also known for its antitumor activity and has hypoglycaemic effects, which was confirmed experimentally in induced diabetes in rats and humans [4]. Antioxidants prevent oxidative damage to the body associated with aging and various diseases such as atherosclerosis, diabetes, cancer and cirrhosis. Oyster mushroom extracts are characterized by antioxidant properties in an experiment with induced rat liver damage. Antioxidants and antioxidant enzymes were significantly increased after delivering of fungal extracts [5]. Similarly, isolated β-glucan had a positive effect on the antioxidant activity of rats and a reduction in induced precancerous colon lesions in rats [6]. The isolates have also been shown to have anti-inflammatory activity as it induces antioxidant and immunomodulatory effects in colitis-induced rats. Furthermore, antibacterial [7], and antifungal activities of these mushrooms have been observed [7]. In the case of *Pleurotus ostreatus*, the antiviral activity of aqueous ethanol extracts has been demonstrated [8,9,10]. Oyster mushrooms have been proven to be a good source of almost all essential amino acids. The concentration of cysteine, methionine and aspartic acid in *P. ostreatus* is higher than in *Agaricus bisporus* and *Lentinula edodes* [11]. Aqueous oyster mushroom extracts contain high concentrations of cysteine, methionine and aspartic acid [12], while methanol extracts help to improve the antioxidant response of the organism and regenerate damaged liver [13]. The aqueous extracts of oyster mushroom brought a remarkable improvement in antioxidant activity in the old rats. Increased protection of the kidneys, brain, liver and heart against oxidative stress due to decreased intensity of lipid peroxidation and enhancement of enzymatic and non-enzymatic antioxidant activities has been observed.

Tchapgang et al. [14] in their recent study of 12 wild mushrooms species found that calcium, magnesium, phosphorus, potassium and sodium were present in all species, while iron was absent in 7 species (*Lactarius gymnocarpus*, *Laccaria longipes*, *Lactarius rubroviolascens*, *Lentinus squarrosulus*, *Termitomyces umkowaan* and *Tylopilus* sp.) in terms of the mineral content of fungal fruiting bodies. In general, mushrooms contained the most phosphorus, potassium and calcium. The iron content was lowest in the fruiting bodies [15]. Furthermore, fungi contain compounds of magnesium, iron, fluorine, copper, manganese, cobalt, titanium and lead [16]. The content of these substances usually increases with the age of the fruiting body. The mineral content depends on the composition of the substrate on which the fungi grow. However, in addition of substances beneficial to health, fungi also absorb some undesirable elements from their surroundings, such as toxic mercury, arsenic, cadmium, chromium, vanadium or beryllium [17,18]. The concentration of these elements in fungi can be several times higher than in the surrounding soil, so it is not recommended in any case to collect fungi in the catchment area of harmful emissions, most often near power plants, aluminum factories or chemical or metallurgical factories [19]. The edges of forests near agricultural land and sites on busy roads can be considered as other risky localities for mushroom harvesting.

Selenium, as another of the accumulated metals, has a significant effect on the function of the human body. For example, it is present in the synthesis of thyroid hormones, in the proper functioning of the body’s immune response as well as in overcoming of oxidative stress. Essentiality of selenium has been proven in 1957. Low concentrations of selenium are effective regarding poisoning with heavy metals such as mercury and arsenic [20]. Previous research shows the possibility of selenium biofortification of garden peas with positive effects on antioxidant content [21]. Selenoergothionein was synthesized in mushrooms growing on substrates artificially enriched with sodium selenate. If selenium is taken into the body, it replaces, together with other compounds, sulphur in ergothioneine. Selenium and zinc-enriched oyster mushroom given to mice had a significant reduction in malondialdehyde levels compared to control mice [22]. Therefore, it is evident that by enriching of cultivated mushroom with inorganic forms of selenium, functional foods can be produced with positive effects on human health, with anti-inflammatory and antitumor effects. Cultivating of saprophytic mushrooms on selenium-rich substrates can be an effective resource of producing selenium-incorporated foods. The selenium content of mushrooms is generally higher than in most vegetables. However, this indication is very variable. The content of selenium in the substrate is decisive. The usual selenium content in the fruiting bodies of fresh edible mushrooms is below the level of <10–200 μg Se. g^−1^ [23].

The aim of the presented study was to determine the content of selected heavy metals in the fruiting bodies of 59 new production strains of oyster mushroom (*Pleurotus ostreatus*), while the concentration of metals was monitored separately in the caps and stipes of the fruiting bodies. Further attention was paid to monitoring of the synergistic and antagonistic relationships of selected metals upon their uptake into the fruiting body. These observations were performed on two types of substrates—namely sodium selenate-free substrate and fortified substrate. The obtained results are important in light of food safety and quality of fruiting bodies. The strain variability characteristic of the species *Pleurotus ostretaus* can be used in the processes of fruiting bodies fortification with selected minerals.

## 2. Materials and Methods

### 2.1. Oyster Mushroom Production Strains

Production strains were acquired through the international cooperation of scientific and educational institutions, mainly from the Czech Republic. A total of 59 production strains of *Pleurotus ostreatus* were used. The known information concerning the origin of the strains is given in Table 1. Some strains could not be specified in more detail, as the records of their isolation have not been maintained.

### 2.2. Setup of the Experiment

The experiments were carried out continuously in the years 2018, 2019 and 2020 during the spring and autumn period. Research tasks with different objectives were solved together as follows:

1. Comparison of the accumulation of individual elements from the point of view of caps vs. stipes,
2. Verification of synergistic and antagonistic relationships in the intake of individual elements on the unfortified variant,
3. Observation of the change of synergistic and antagonistic relationships in the intake of individual elements after substrate fortification with sodium selenate.

### 2.3. Growing of Biological Material

Biomass of selected production strains intended for monitoring the accumulation of individual elements in different parts of the fruiting body was grown in controlled conditions of a growing unit. Unfortified substrate (without selenate) was composed of pressed wheat pellets, which are intended for use as bedding for horses. An identical production batch was used. The pellets were put into transparent plastic containers and were watered with cold drinking water in a ratio of 1 part of pellets and 2.6 parts of water volume. After swelling, prepared substrates were incubated at 25 °C for 48 h and then subsequently at 60 °C for 24 h. All competing microorganisms were suppressed by this process.

**Figure 1:**
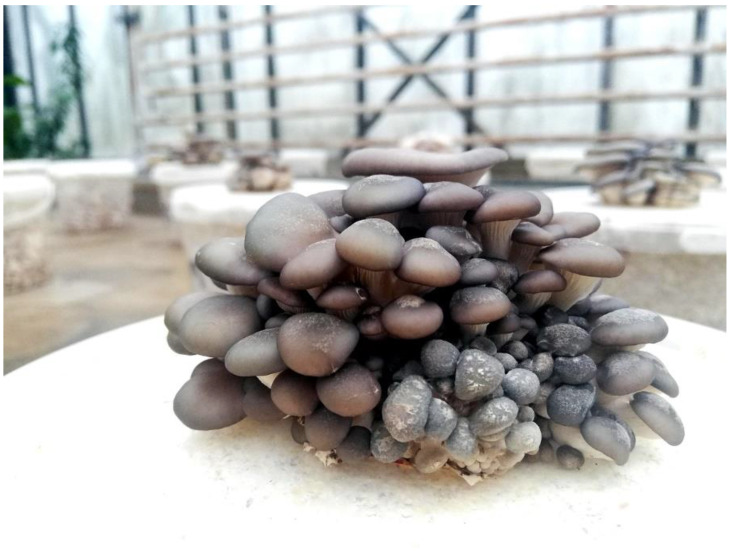
Fruiting body of *Pleurotus ostreatus* (Source: Author of the work).

**Figure 2:**
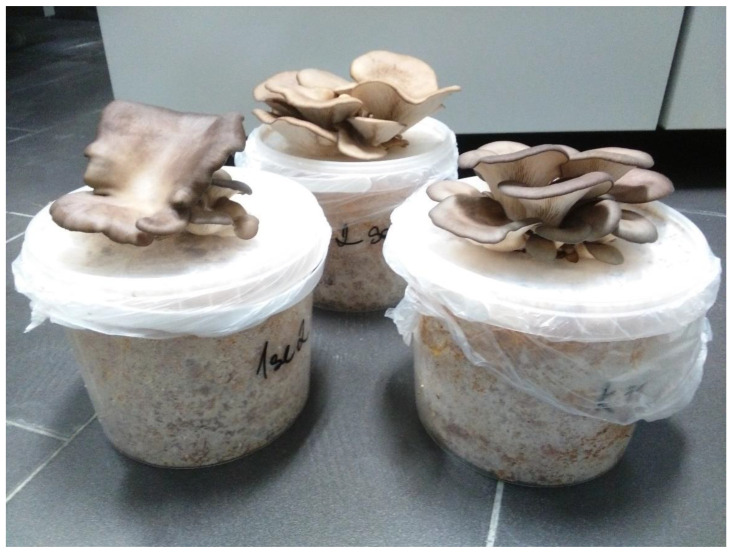
Fruiting body of *Pleurotus ostreatus* (Source: Author of the work).

The inoculation and the incubation of mycelia were performed at 25 °C in a dose of 5% inoculum of the selected strains. The production of the fruiting bodies took place under the controlled conditions with a daily maximum of 16 °C, when alternating between day and night. A minimum night temperature of 5 °C was ensured during the night (Figure 1). Also, high relative humidity and CO_2_ ventilation were controlled (Figure 2). The collection took place gradually, over a period of several weeks. After the harvest, the fruiting bodies were divided into stipe (s) and cap (c) and dried in a laboratory hot air dryer Memmert UF 110 Plus (Memmert, Schwabach, Germany) at 45 °C. Subsequently, they were milled in shear mill Retsch SM 100 (Retsch, Haan, Germany) and stored until the use. A total of 59 variants with three replicates were established. The experiment was repeated in 2 growing seasons (spring and autumn) for 2 years (2018, 2019).

Fortified substrate (with selenate) was produced, and fruiting bodies were grown under almost identical conditions as described at the beginning of the chapter 2.3. In contrast to the previous substrates, the drinking water for the establishment of the substrates was replaced by a fortification solution of sodium selenate with 0, 0.5, 1.0 and 2.0 mg dm^−3^ Se. P.O. strain 2 was randomly selected as the model strain for this research task. This strain is common in the practice. After the harvest, the fruiting bodies were lyophilized (Telstar LYOQUEST -55 (Azbil Telstar Technologies S. L. U., Barcelona, Spain).) and then milled and stored. The experiment consisted of 4 variants and each variant contained 10 replicates. The experiment was repeated in 2 growing seasons (spring and autumn) for 2 years (2019, 2020).

### 2.4. Selenium and Selected Hazardous Metals Determination

Selenium—The plant material was mineralized in the microwave mineralizer type “CEM Mars X” (microwave digestion oven) followed by weighing of a 0.5 g of sample in the mineralization container. After wetting with 1 mL double distilled water, 5 mL of conc. HNO_3_ and 1 mL of H_2_O_2_ was added. The product was mineralized at 150 °C for 20 min and then refilled into volumetric flask till 25 mL. ET-AAS method with Zeeman-effect background corrections have been applied to reduce spectral interference in case of quantitative selenium analysis. The total selenium content was estimated by using of atomic absorption spectrometer SpectrAA240FS (Varian, Mulgrave, Australia). The operating conditions were as follows: cathode selenium lamp, current 10 mA, wavelength 196 nm, slit width of 1.0 nm. The graphite cuvette heated at 2600 °C was used as the atomizing medium. Sample injection volume was 10 μL. Palladium modifier Pd (NO_3_)_2_ with a concentration of 0.1 mol dm^−3^ and 1% ascorbic acid was used as the modifier. Calibration curve was used for determination of tested compounds concentration in an aqueous solution [24].

The following validation parameters were determined: Precision (under repeatability conditions), trueness, limit of detection, limit of determination.

Repeatability of the method was characterized by selected variation coefficient Sr, calculated from standard deviation s and arithmetic average from a series of measurements under repeatability conditions. Repeatability of the method: 6.2%.

Trueness of the method was verified by the analysis of matrix reference material SRM 1570a (NIST, Gaithersburg, MD, USA)—spinach leaves. Recovery of the method: 95.2%. Limit of detection and limit of determination are calculated from the upper limit approach (ULA) [25]. The condition is equidistant distribution of concentrations. Relation for determination limit calculation LOQ (limit of quantitation) = 3 × LOD (lower limit of detection). LOD = 0.0028 mg kg^−1^ Se, LOQ = 0.0084 mg kg^−1^ Se DM (dried matter). Calibration curve: linear. Linearity of the method was evaluated as an ability to provide results proportional to concentration within the defined interval.

Minerals—The analysis of selected elements concentration was performed by inductively coupled plasma optical emission spectrometry on a dual ICP-OES iCAP7600 instrument (Thermo Scientific, Waltham, MA, USA). An ETHOS UP instrument (Milestone, Sorisole, Italy) was used for microwave mineralization of samples in a mixture of 5 mL Please explain abbreviation

HNO_3_ and 2 mL H_2_O_2_. Increasing of the temperature in to 200 °C lasted 15 min, the temperature of 200 °C was maintained for 15 min, and cooling for 30 min. The power of the radio frequency transmitter was 1150 W, the gas flow through the nebulizer 0.45 L/min, and the cooling gas flow 12 L/min, auxiliary gas flow rate of 0.5 L/min. The exposure time at UV wavelengths was 15 s, and at VIS wavelengths 5 s. The samples were measured three times. The basic validation characteristics are given in Table 2.

Precision was determined by repeated measurements of a real matrix sample, expressed as RSD (%). Detection limits (LOD) and quantification limits (LOQ) were calculated from the BEC (Background Equivalent Concentration) value determined from the condition of the analytical signal intensity ratio and the background intensity. A mixed standard of elements Al, Ag, Ba, Be, Bi, Ca, Cd, Co, Cr, Cs, Cu, Fe, Ga, In, K, Li, Mg, Mn, Mo, Na, Ni, Pb, Rb, Sr, Tl, V, Zn (Multielement standard solution V for ICP, SigmaAldrich, St. Louis, MO, USA) was used for calibration of ICP-OES (Inductively coupled plasma—optical emission spectrometry). The working gas for ICP-OES was argon with a purity of 99.999% (Messer Tatragas, Bratislava, Slovakia).

Mercury was determined on a single-purpose atomic absorption spectrometer AMA 254 (Altec, Prague, Czech Republic), which is intended for direct determination of mercury content in solid and liquid samples without chemical sample pre-treatment. It uses a technique of generating metallic mercury vapours and then capturing them on a gold amalgamator, which results in an extremely high assay sensitivity.

Validation parameters: Precision (under repeatability conditions), trueness, limit of detection, limit of determination.

Repeatability of the method was characterized by selected variation coefficient Sr, calculated from standard deviation s and arithmetic average from a series of measure-ments under repeatability conditions. Repeatability of the method: 3.40%.

Trueness of the method was verified by the analysis of matrix reference material SRM 1570a (NIST, Gaithersburg, MD, USA)—spinach leaves. Recovery of the method: 95.2%.

Limit of detection and limit of determination were calculated from the blank measurement. LOD = average noise value + 3 × SD (Standard deviation). Relation for determination limit calculation LOQ = 3 × LOD. LOD = 0.00020 mg kg^−1^ Hg, LOQ = 0.00060 mg kg^−1^ Hg DM. Calibration curve: linear. Linearity of the method was evaluated as an ability to provide results proportional to concentration within the defined interval.

### 2.5. Statistic Analysis

Data were analyzed by using the Statgraphics Centurion XVII (Statgraphics Technologies, Inc., The Plains, VA, USA) software with the technique for analyzing of categorical factors effect—Analysis of Variance—ANOVA, the LSD (Least Significant Difference) test and Multiple variable analysis—Pearson product moment correlations between each pair of variables.

## 3. Results and Discussion

### 3.1. The Cumulation of Elements in the Individual Parts of the Fruiting Body

Mineral elements play an important role in metabolism as they are needed for various metabolic responses, formation of rigid bone, water regulation and saline balance, sensory stimulation and other functions [26]. According to Singh, A. and Singh S. [27] oyster mushroom has mineral composition which is highly valuable because of the content of numerous microelements.

In our research, the content of aluminum (Al), barium (Ba), calcium (Ca), copper (Cu), iron (Fe), potassium (K), magnesium (Mg), manganese (Mn), sodium (Na) and zinc (Zn) were determined in samples of the stipes and the caps of various strains of *Pleurotus ostreatus*. The average values of the selected substances in the stipes and in the caps are shown in the Table 3. The Table 4 and Table 5 shows the lowest and the highest measured values of selected substances in the production strains of *Pleurotus ostreatus* samples.

The concentration of the metal elements can vary in different types of mushrooms. Gogavekar et al. [28] claimed in their study that the mean metal concentration in *P. ostreatus* fruiting bodies was in the order: Ca > Fe > Mg > Na > K > Zn > P > Ni > Mn > Pb > Cu > Cr > Co. In our study the mean metal concentration was slightly different compared to the results from Gogavekar et al. [28]. We found out that the mean element concentration in the stipe of *P. ostreatus* was in the order: K > Mg > Ca > Na > Fe > Zn > Cu > Al > Ba > Mn. The mean element concentration in the cap of *P. ostreatus* was in the following order: K > Mg > Ca > Na > Zn > Fe > Mn > Cu > Al > Ba.

In many scientific papers it is written that many fungi can absorb certain metals and metalloids from their substrate into their fruiting bodies in high concentrations [29,30,31]. Zakil et al. [32], in their study, had 5 different substrate combinations with various biomass ratios which were used for cultivation of *P. ostreatus*. As a substrate for cultivation of *P. ostreatus* was used oil palm empty fruit bunch, oil palm press fiber, oil palm frond, sugarcane bagasse and corn cob. The aim of the study was to contrast the impact of various agricultural wastes on the development, yield and mineral content of *P. ostreatus*. The content of selected macronutrients (Ca, Mg, P, K) and micronutrients (Na, Fe, Zn, Mn) was analyzed. Comparing our results of the selected substances with the results of the study by Zakil et al. [32], we found out that a significantly higher concentration of Ca, Fe and Mn was observed in *P. ostreatus* samples from Zakil et al. [32]. On the other hand, it was also found out that in our analyzed samples there was a higher content of Mg, K, Na and Zn than in the monitored samples from Zakil et al. [32]. The authors of Regula et al. [33], however, in their study, grew fruiting bodies on the same substrate as in our study (wheat straw), and they reported related concentrations of metallic elements. The authors of Mleczek et al. [15] in their research state that the fungi *Pleurotus ostreatus*, *Lentinula edodes*, and especially *Agaricus bisporus*, can be a valuable source of macro- and micronutrients such as K, P, Cu, Fe and Se. Previously mentioned types of mushrooms also contain significantly high levels of K relative to Na, which advocate their potential use as foods to reduce the Na/K ratio. Their findings from the research correspond with ours. However, the detected concentrations of metals, which were published by the authors, for a particular fungal species vary considerably. The reason is the great diversity of growing substrates as well as the production strains used.

Calcium belongs to essential minerals. In humans, calcium is an essential component in the prevention and development of osteoporosis and in the formation of strong bones and teeth [34]. In the study from Patil et al. [35] the *P. ostreatus* was cultivated on the different types of the substrates, and it was found that Ca content ranged from 2400 mg kg^−1^ to 3300 mg kg^−1^. The content of Ca in *P. ostreatus* cultivated on the wheat straw was 2700 mg kg^−1^. Comparing our results with the results from Patil et al. [35], related concentrations of Ca were observed.

Riaz and Guerinot [36] state that Fe is one of the essential micronutrients which is required by plants and animals. According to Raman et al. [37] about 90% of the bioavailability of Fe in the edible mushroom is easily absorbable. The result in our study shows that the stipe of *P. ostreatus* contained on average 40 mg kg^−1^ of Fe (SD ± 21, n59) and the cap of *P. ostreatus* contained on average of 56 mg kg^−1^ of Fe (SD ± 23; n59). Raman et al. [37] claim that in *Pleurotus* species content of Fe has been reported in the range of 5.5–13.4.

Budzyńska et al. [38] in their research were verifying a possible interaction between Fe and Ca and they were estimating the role of the addition to stimulate Fe accumulation in *Pholiota nameko*. Results of the research done by Budzyńska et al. [38] show that when the Fe concentration was higher in the substrate, while also a significantly higher accumulation of Fe was in *P. nameko*. Also, it was found that the presence of Fe in the substrate may promote accumulation of the other elements, such as K, Mg, Mn, Na, P and S. Although the addition of may promote the accumulation of the previous mentioned elements, the addition of Ca stimulates and/or inhibits their content in fruiting bodies. The research also pointed out the synergism between Fe and Ca, where the addition of Ca stimulated Fe accumulation and the concentration of Fe in the substrate stimulated Ca accumulation.

Singh et al. [27] state that the content of Cu in *Pleurotus* mushroom was found higher (12.2 to 21.9 mg kg^−1^) as compared to another mushroom. In our study the stipe contained on average 5.3 mg kg^−1^ of Cu (SD ± 2.4, n59), where the lowest measured content of Cu in the stipe was 1.7 mg kg^−1^ (P.O. strain 29) and the highest measured content of Cu in the stipe was 12.0 mg kg^−1^ (P.O. strain 38). The cap contained on average 6.2 mg kg^−1^ of Cu (SD ± 1.5, n59), where the lowest measured content of Cu in the cap was 3.0 mg kg^−1^ (P.O. strain 36) and the highest measured content of Cu in the cap was 12.0 mg kg^−1^ (P.O. strain 39). Authors Sanglimsuwan et al. [39] observed that from 21 analyzed mushroom samples, *Pleurotus ostreatus* was the most resistant to the high concentrations of copper, cadmium, zinc, nickel, cobalt and mercury in the substrate.

There are large number of scientific studies that prove that edible mushrooms can be a significant accumulator of toxic compounds. Aluminium is one of the toxic elements [40]. In mushrooms its concentration can vary singnificantly [40]. In our study the average value of Al in the stipe was 4.9 mg kg^−1^ Al (SD ± 4.5, n31) and in the cap the average value was 5.5 mg kg^−1^ Al (SD ± 4.6, n19). Wesołowska et al. [41] studied concentration of various metals in *Xerocomus badius* (Fr.), *Suillus luteus* (L.) and *Leccinum scabrum* (Bull.) Grey) mushrooms in different distance from the border of an industrial area. It was reported that concentration of Al ranged from 2.8–39.6 mg kg^−1^ dry matter and significantly (*p* < 0.05) decreased with increasing distance from the industrial plant. From all studied in the study from Wesołowska et al. [41], only the concentration of Al in the tested samples was distance dependent. From the above, it can be stated that accumulation of some metals in the fruiting bodies may be caused by the environmental pollution near metallurgical plants.

Rózsa et al. [42] state that Mg is a mineral substance which plays an important role in oxidation processes. In our research, it was detected in range concentration of Mg in the stipe from 880 mg kg^−1^ to 2400 mg kg^−1^ and in the cap the range of Mg concentration from 1400 mg kg^−1^ to 2400 mg kg^−1^. The concentration of Mg in the samples of *P. ostreatus* in the study by Zakil et al. [32] ranged from 270 mg kg^−1^ to 1140 mg kg^−1^. The research done by Włodarczyk et al. [43] found that addition of inorganic Zn and Mg salts into the media resulted in the increase of the production biomass by 30% and in the increase of bioaccumulation of the inorganic salts.

According to Patil et al. [35] concentration of Na of *Pleurotus ostreatus* was variable with different substrates. In a study from Patil et al. [35] the range of Na concentration was recorded from 2600 mg kg^−1^ to 3100 mg kg^−1^, but concentration of Na in *P. ostreatus* cultivated on wheat straw was 3050 mg kg^−1^. In our study we had mean Na content in the samples of *Pleurotus ostreatus* in the stipe 590 mg kg^−1^ and in the cap 600 mg kg^−1^. In the research by Zakil at al. [32] it was mentioned that only in one type of substrate was detected the content of Na in *P. ostreatus* and its content was 67 mg kg^−1^. When comparing the results with previously mentioned studies, it can be stated that the content of Na in *Pleurotus ostreatus* samples in our study is higher.

Potassium is a mineral element which has an important role in metabolism [44]. According to published data in the literature, it is stated that concentration of K in the mushroom samples was between 19,000 and 54,073 mg kg^−1^ [45,46]. In our study, it was measured that mean concentration of K in the *P. ostreatus* samples in the stipe was 26,000 mg kg^−1^ and in the cap the mean concentration of K was 34,000 mg kg^−1^. Based on these values, it was concluded that obtained values were compatible with the literature data.

Mn is a substance which occurs naturally in nature [44]. In our research, the lowest detected content of Mn in the stipe was 0.39 mg kg^−1^ (P.O. strain 36) and the highest detected content of Mn in the stipe was 4.8 mg kg^−1^. The lowest detected content of Mn in the cap was 2.8 mg kg^−1^ (P.O. strain 45) and the highest detected content of Mn in the cap was 11.0 mg kg^−1^ (P.O. strain 34).

Zinc is classified as a trace element which is found in biological fluids. Zinc is a necessary substance in several enzymatic processes, in DNA synthesis, in material transitions in biological membranes and in the immune system [44]. The data from our results shows that the lowest measured content of Zn in the stipe was 11 mg kg^−1^ (P.O. strain 45) and the highest measured content of Zn in the stipe was 83 mg kg^−1^ (P.O. strain 31). The lowest measured content of Zn in the cap was 140 mg kg^−1^ (P.O. strain 45) and the highest measured content of Zn in the cap was 920 mg kg^−1^ (P.O. strain 1). The concentration of Zn in the samples of *P. ostreatus* in the study by Zakil et al. [32] ranged from 17 mg kg^−1^ to 48 mg kg^−1^. Based on these values, it may be concluded that the values in our study were higher than data from Zakil et al. [32].

It was observed that in the stipe there was significantly higher content of barium (Ba) than in the cap. The stipe contained on average 4.8 mg kg^−1^ Ba (SD ± 1.3, n56), where the lowest detected content was 3.0 mg kg^−1^ Ba (P.O. strain 5), and the highest detected content was 9.7 mg kg^−1^ Ba (P.O. strain 39). The cap contained an average of 4.0 mg kg^−1^ Ba (SD ± 0.96, n56), where the lowest detected content was 2.6 mg kg^−1^ Ba (P.O. strain 17), and the highest detected content was 6.8 mg kg^−1^ Ba (P.O. strain 26).

The acceptability of metals by plants depends on many factors, e.g., chemical form in which the metal is bound, its solubility, etc. Important is the pH of the growing medium. Most metals in neutral to alkaline environments are not available to plants. In general, in mildly acidic and acidic substrates, at pH values > 5, the metals get into an acceptable form for plants. Heavy metals can be taken up by plants passively or actively by root cells. The ions in the soil solution reach the surface of the roots and penetrate the root cells. It is believed that plants have a special mechanism to detoxify the metal. Plants store metals in cell walls or vacuoles or convert the inorganic form of the metal inside the cell to a less harmful form, most often an organic complex. Probably, peptide-binding compounds—phytochelatins, which occur in plants growing at high metal concentrations, protect plant cells from damage. Analyses of various plant organs have made possible to determine the functional relationship between metal uptake, translocation and accumulation. It has been found that different ways of metal uptake are associated with different plant tolerance and metal toxicity. Excluders limit the uptake and transport of metal by immobilizing it in the root. At different concentrations of metals in the soil, the above-ground parts of the plants have a relatively low content. The metal content in the plant accurately reflects the external environment content of the indicator plants. Accumulators are plants, which, thanks to specialized physiology, actively concentrate metals in the aboveground parts. The metals are stored in leaves in vacuoles. Hyperaccumulators, which also include mushrooms, actively concentrate metals in the above-ground parts, where they reach significantly higher values than in the roots and soils [47]. The essence of heavy metal toxicity to plants lies in their high affinity for chemical groups containing reduced forms of sulfur, so that they deactivate SH—enzymes) [48,49].

As with plants, fungi are known to have a tendency to accumulate different substances from the substrate and the environment, while hazardous metals are not an exception. At least four factors can affect the concentrations of elements in edible fungi, which are: species, ecology (saprophyte, decaying and mycorrhizal wood), morphological parts (cap, hymenophore, stipe, mycelium, etc.) and physical properties of the soil (e.g., metals level, pH, and composition of soil) [50]. From the point of view of the consumer, mycoremediation and pharmaceuticals, the occurrence of minerals in specific parts of the fruiting body can be very useful information, especially when it is such a commercially interesting mushroom as oyster mushrooms.

The analysis of the measured values has shown that a statistically significant difference between selected substances content in the stipe and the cap was confirmed at Cu, Fe, K, Mg, Mn and Zn, where higher content of these substances was determined in the caps than in the stipes. A significantly higher content of Ba was observed in the stipe than in the cap. No significant statistical difference between the content in the stipes and in the caps was observed at Al, Ca and Na.

The different distribution of elements was also mentioned in the publication from Kalač [51] where he states that potassium has an uneven distribution in the fruiting bodies and its content decreases in the following direction: cap (the highest content)-stipe-spore-forming part-spores (the lowest content). In our study, comparable results were recorded where the K content was significantly higher in the cap then in the stipe.

Despite the very good availability of information about the topic of the biosorption of fungi, there is little published in the scientific databases about which parts of the fruiting body the individual metals dominate. In the Table 3 we present the average values of the content of selected substances in the stipe and in the cap.

### 3.2. Synergistic and Antagonistic Relationships in Unfortified Variants

**Figure 3:**
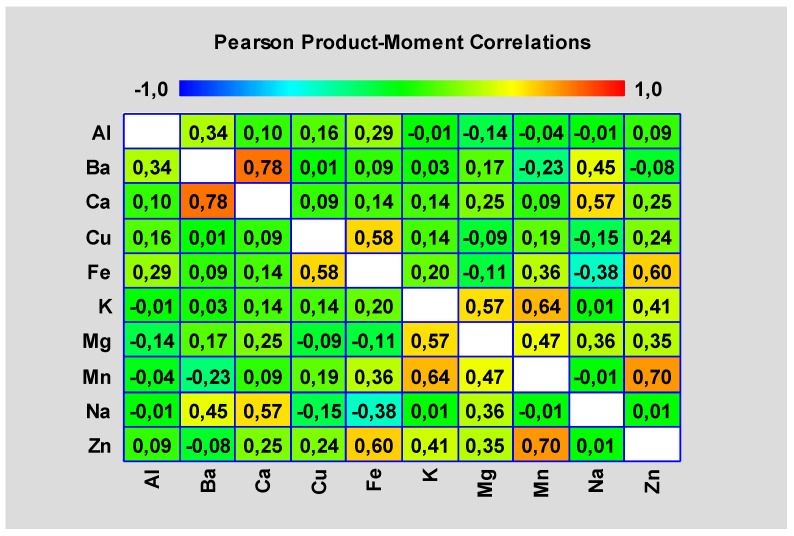
Pearson product moment correlations—unfortified variant (Source: Author of the work).

In an unfortified variant by selenium, the interaction of 10 elements was evaluated. Specifically, the elements: aluminum, barium, calcium, copper, iron, potassium, magnesium, manganese, sodium and zinc were taken in account. Figure 3 shows Pearson product moment correlations between each pair of variables. These correlation coefficients range between −1 and +1 and measure the strength of the linear relationship between the variables.

Statistically significant non-zero correlations at the 95.0% confidence level (*p* < 0.05) are Al and Ba, Al and Fe, Ba and Ca, Ba and Na, Ca and Na, Cu and Fe, Fe and Mn, Fe and Na, Fe and Zn, K and Mg, K and Mn, K and Zn, Mg and Mn, Mg and Na, Mg and Zn and Mn and Zn.

#### 3.2.1. Synergistic Relationships

Statistically significant (*p* < 0.05) synergistic relationships were shown in pairs Al and Ba, Al and Fe, Ba and Na, Ba and Ca, Ca and Na, Cu and Fe, Fe and Mn, Fe and Zn, K and Mg, K and Mn, K and Zn, Mg and Mn, Mg and Na, Mg and Zn and Mn and Zn.

Various synergistic relationships were also detected by Raiesi and Sadeghi [52] and Luo and Rimmer [53]. They claim that Pb and Ni have a synergistic effect on Cd translocation and accumulation. If Cd co-occurs with Ni, the absorption of Cd is stronger. Cu also increases the toxicity of Zn in barley.

#### 3.2.2. Antagonistic Relationships

The antagonistic relationships were detected only for the Fe and Na variant.

### 3.3. Synergistic and Antagonistic Relationships in Variants Fortified with Selenium

Fungi of edible mushrooms are used in pharmaceuticals, biotechnologies but mainly in gastronomy. Their texture, taste, chemical and nutritional properties are important [54]. The risk of their use may be the accumulation of several trace elements, especially mercury, cadmium and lead and metalloids—namely, arsenic and radionuclides [55].

During our experiment we evaluated the content of 11 metal elements (copper, zinc, manganese, iron, cobalt, nickel, chromium, lead, cadmium, mercury and selenium), including some toxic ones (nickel, chromium, lead, cadmium and mercury). The average values for the individual variants of the first and second cultivation period are shown in Table 6.

**Figure 4:**
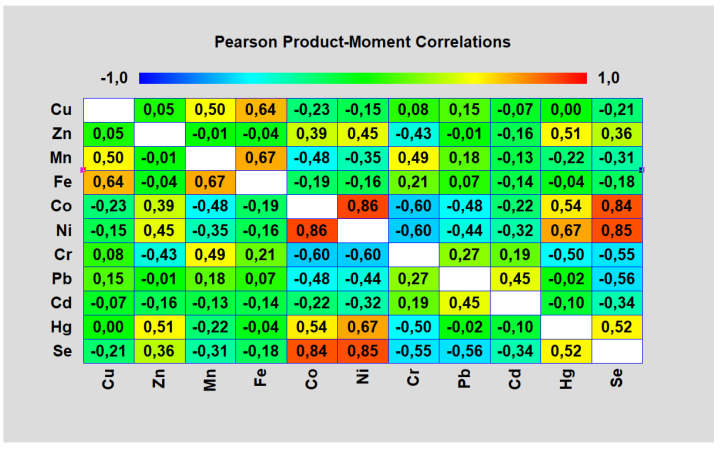
Pearson product moment correlations-selenium fortified variant (Source: Author of the work).

Figure 4 shows Pearson product moment correlations between each pair of variables. These correlation coefficients range between −1 and +1 and measure the strength of the linear relationship between the variables.

*p*-values below 0.05 indicate statistically significant non-zero correlations at the 95.0% confidence level. The following pairs of variables have *p*-values below 0.05. Cu and Mn, Cu and Fe, Zn and Co, Zn and Ni, Zn and Cr, Zn and Hg, Zn and Se, Mn and Fe, Mn and Co, Mn and Ni, Mn and Cr, Co and Ni, Co and Cr, Co and Pb, Co and Hg, Co and Se, Ni and Cr, Ni and Pb, Ni and Cd, Ni and Hg, Ni and Se, Cr and Hg, Cr and Se, Pb and Cd, Pb and Se, Cd and Se and Hg and Se.

#### 3.3.1. Synergistic Relationships

Specifically, in relation to the applied selenium, it was possible to observe significant (*p* < 0.05) synergism with elements Ni, Co and Hg. In the control variant 0.68 mg kg^−1^ Ni DM, 0.61 mg kg^−1^ Co DM and 0.041 mg kg^−1^ Hg DM were detected. In the variant with 0.5 mg dm^−3^ Se an increase in nickel content about 66.2%, cobalt about 49.2% and mercury about 15.25% was observed, in the variant with 1.0 mg dm^−3^ Se nickel about 136.8%, cobalt about 62.3% and mercury about 15.60% and in the variant with 2.0 mg dm^−3^ Se an increase in nickel concentration about 170.6%, cobalt about 103.3% and mercury about 15.96% was detected.

The synergism of selenium with zinc has not been statistically proven (*p* < 0.05). After application of 0.5 mg dm^−3^ Se there was about 2.7% increase. After further increasing of the selenium content in the growing substrate (1.0 and 2.0 mg dm^−3^ Se) the increase was approximately equal—4.8% and 4.7%, respectively.

After the application of sodium selenate, other synergistic reactions were observed between Cu and Mn, Cu and Fe, Zn and Co, Zn and Ni, Zn and Hg, Mn and Fe, Mn and Cr, Co and Ni, Co and Hg, Ni and Hg, Pb and Cd.

Some authors suggest that there is a positive correlation between cadmium, copper and zinc intake [56,57]. However, this relationship was not demonstrated in our study after selenium application. As we mentioned before, the cumulation is affected by physics and chemical properties of soil, e.g., concentration levels of some metals [50]. This may explain the unproven correlation between our findings and the findings of other authors [56,57].

Other authors monitored 18 species of wild mushrooms [58]. They confirmed significant correlations between chromium and nickel (+0.836), chromium and manganese (+0.546), chromium and zinc (+0.664), nickel and manganese (+0.618) and manganese and zinc (+0.616). These findings are only partially consistent with our results. The differences are caused by diversity of the growing substrates, which, in each substrate, dominated a different element.

#### 3.3.2. Antagonistic Relationships

Statistically proven (*p* < 0.05) Se antagonism was observed with the uptake of Mn, Cr, Pb and Cd elements. The control variant contained 8.20 mg kg^−1^ Mn DM (Table 6), with an increase in selenium doses in the substrate (0.5, 1.0 a 2.0 mg dm^−3^ Se) a decrease in manganese content from 4.6% to 6% was observed. A significant decrease in chromium content occurred proportionally with increasing selenium concentration in the substrate from 15.7% to 47.2%. The lowest applied selenium dose (0.5 mg dm^−3^ Se) statistically significantly increased lead and cadmium intake by fruiting bodies (about 19.8% Pb, 4.0% Cd), but higher selenium concentrations significantly reduced lead accumulation about 35.2% and cadmium about 24.0% (2.0 mg dm^−3^ Se).

Statistically unproven (*p* < 0.05) Se antagonistic relationships were observed in the accumulation of Cu and Fe elements. The control variant contained 7.50 mg kg^−1^ Cu DM and 49.0 mg kg^−1^ Fe DM (Table 6). With increasing selenium doses in the substrate (0.5, 1.0 and 2.0 mg dm^−3^ Se) decreases in copper content from 1.5% to 5.0% and iron from 1.0% to 3.9% were observed.

Other antagonistic relationships between Mn and Co, Mn and Ni, Co and Cr, Co and Pb, Ni and Cr, Ni and Pb, Ni and Cd and Cr and Hg were present after selenium application.

The authors Pavlik at al. [59] also consider in their study that the content of individual compounds in fruiting bodies varies significantly depending on the type and composition of the substrate as well as the production strain of the mushroom. The aim of their study was to grow fruiting bodies on waste ash from the heating plant. In their work, they found that the control sample of fruiting bodies of strain X12 without ash fortification contained 5230 mg kg^−1^ P, 4880 mg kg^−1^ Ca, 2830 mg kg^−1^ Mg, 4730 mg kg^−1^ K, 235 mg kg^−1^ Na, 0.28 mg kg^−1^ Mn, 0.12 mg kg^−1^ Fe, 145 mg kg^−1^ B, 8.47 mg kg^−1^ Al, 6.59 mg kg^−1^ Cu, 73.30 mg kg^−1^ Zn, 0.14 mg kg^−1^ Cd and 1.44 mg kg^−1^ Pb. A sample of strain X12 fortified with wet ash contained 3160 mg kg^−1^ P, 55,000 mg kg^−1^ Ca, 4640 mg kg^−1^ Mg, 4720 mg kg^−1^ K, 1459 mg kg^−1^ Na, 10.90 mg kg^−1^ Mn, 16.60 mg kg^−1^ Fe, 402 mg kg^−1^ B, 58.20 mg kg^−1^ Al, 41.70 mg kg^−1^ Cu, 79.90 mg kg^−1^ Zn, 0.27 mg kg^−1^ Cd and 0.23 mg kg^−1^ Pb. For strain number 184, the control sample of fruiting bodies contained 7660 mg kg^−1^ P, 61,700 mg kg^−1^ Ca, 4030 mg kg^−1^ Mg, 7270 mg kg^−1^ K, 312 mg kg^−1^ Na, 0.39 mg kg^−1^ Mn, 0.32 mg kg^−1^ Fe, 189 mg kg^−1^ B, 8.68 mg kg^−1^ Al, 8.19 mg kg^−1^ Cu, 116 mg kg^−1^ Zn, 0.21 mg kg^−1^ Cd and 1.17 mg kg^−1^ Pb. A sample of strain 184 fortified with wet ash contained 3220 mg kg^−1^ P, 59,100 mg kg^−1^ Ca, 4840 mg kg^−1^ Mg, 5530 mg kg^−1^ K, 1166 mg kg^−1^ Na, 13.60 mg kg^−1^ Mn, 19.10 mg kg^−1^ Fe, 472 mg kg^−1^ B, 82.60 mg kg^−1^ Al, 50.30 mg kg^−1^ Cu, 101 mg kg^−1^ Zn, 0.40 mg kg^−1^ Cd and 0.07 mg kg^−1^ Pb. The published results can be best explained, as in our work, by the presence of synergistic and antagonistic relationships in the uptake of individual elements into the fruiting bodies. The strong concentration of some elements in the growing substrate suppresses the accumulation of other elements in the fruiting bodies and vice versa.

A similar principle of accumulation works also in case of different unfortified substrates. The authors Koutrotsios et al. [60] found that fresh oyster mushroom fruiting bodies grown on grape pomace with cotton content contained 0.07 mg kg^−1^ As, 15.53 mg kg^−1^ Be, 170 mg kg^−1^ Ca, 0.38 mg kg^−1^ Cd, 0.03 mg kg^−1^ Co, 21.13 mg kg^−1^ Cu, 0.09 mg kg^−1^ Fe, 0.61 mg kg^−1^ Li, 2400 mg kg^−1^ Mg, 11.06 mg kg^−1^ Mn, 0.09 mg kg^−1^ Mo, 0.52 mg kg^−1^ Ni, 0.21 mg kg^−1^ Sb, 0.21 mg kg^−1^ Se, 1.25 mg kg^−1^ Sr and 118.26 mg kg^−1^ Zn, while fruiting bodies grown with the same technology but on a substrate composed from almond shells and walnuts in a ratio of 1: 1 contained 0.03 mg kg^−1^ As, 6.45 mg kg^−1^ Be, 0.73 mg kg^−1^ Ca, 0.38 mg kg^−1^ Cd, 0.03 mg kg^−1^ Co, 39.05 mg kg^−1^ Cu, 0.13 mg kg^−1^ Fe, 0.29 mg kg^−1^ Li, 0.28 mg kg^−1^ Mg, 13.76 mg kg^−1^ Mn, 0.19 mg kg^−1^ Mo, 0.69 mg kg^−1^ Ni, 0.35 mg kg^−1^ Sb, 0.34 mg kg^−1^ Se, 2.57 mg kg^−1^ Sr and 110.41 mg kg^−1^ Zn. Based on the above, it can be stated that it is possible to use an admixture of some natural materials to fortify the substrates.

As reported by Rashid et al. [61], great emphasis needs to be placed on the geographical origin of the substrate and the related quality. The authors report that while cadmium in *Pleurotus ostreatus* was 5.39 mg kg^−1^ Cd (DM) in fruiting bodies harvested in Mexico, 0.41 mg kg^−1^ Cd (DM) was found in Bangladesh and only 0.074 mg kg^−1^ Cd (DM) in Brazil. The review talks about the different quality of local substrates. Industry and environmental pollution significantly affect the quality of substrates. Substrates must be monitored for contaminants. In the case of chromium, the concentration in fruiting bodies was 63.0 mg kg^−1^ Cr (DM) in Mexico and 0.30 mg kg^−1^ Cr (DM) in Bangladesh. Other authors [58] monitored commercially marketed mushroom fruiting bodies from retail chain counters and found that the analyzed fruiting bodies were not risky for the consumer. They were detected 52.9 mg kg^−1^ Fe, 34.5 mg kg^−1^ Zn, 1.28 mg kg^−1^ Cu, 0.143 mg kg^−1^ Ar, 0.095 mg kg^−1^ Cd, 0.021 mg kg^−1^ Pb and 0.0022 mg kg^−1^ Cu in fresh fruiting bodies. The substrate used to produce these fruiting bodies was of good quality. However, if it is not possible to use a substrate of satisfactory quality, or only a substrate with higher levels of hazardous metals is available, we recommend using fortification of the substrate with, for example, sodium selenite, to create synergistic and antagonistic relationships. In this way it is possible to eliminate the intake of some risk elements.

## 4. Conclusions

The penetration of heavy metals into plants is influenced by soil ecological condi-tions, such as soil types, soil pH, concentration and form of heavy metals, humus content in soil, oxidation-reduction conditions around the root system associated with microbial processes of organic matter decomposition, moisture, temperature, soil compaction, used fertilizers and plant protection products. Although mushrooms, unlike plants, are not chemoautotrophic organisms, many factors affecting the accumulation of heavy metals are identical. Various studies show that, despite the above factors, the accumulation of risk metals from the substrate is significantly affected by species and strain variability of the model organism. Significant differences between the observed strains were found in the work. According to this fact the possibility of using less cumulative strains for substrates with a high content of heavy metals from specific locations around the world was confirmed. From the perspective of the distribution of selected metals in the fruiting body, differences were found. More Cu, Fe, K, Mg, Mn, Zn accumulated in the cap, while in the stipe Ba had amassed. The ability of oyster mushroom fruiting bodies to accumulate different metals from the substrate was confirmed, as well as the large variability of the accumulation potential of different strains of the same species. Published results may be helpful in the production of fortified foods (fruiting bodies, mushroom powder) as well as in the mycoremediation environment (for example P.O. strain 39 and P.O. strain 26 for Barium. In the production of fortified fruiting bodies by selected mineral substances (for example Zn), it is possible to identify and use specific hypercumulative production strains (for example P.O. strain 3 and P.O. strain 11).

During the research, synergistic and antagonistic relationships between the observed metals were demonstrated. After the application of selenate, these relationships were affected and changed. We confirmed the conclusions of other authors who claim that an excess of one metal in the substrate significantly affects the uptake of another metal.

## Figures and Tables

**Table 1:** Biological material/strains of *Pleurotus ostreatus*.

Designation	Identificaton	Description
P.O. strain 1	HK35	dr. Jablonský, Czech University of Life Sciences Prague
P.O. strain 2	Kryos B	dr. Jablonský, Czech University of Life Sciences Prague
P.O. strain 3	P-80	Mr. Rajtár, Mycoforest Company Slovakia,
P.O. strain 4		dr. Pavlík, Zvolen, spruce harvest
P.O. strain 5	2175	Mr. Rajtár, Mycoforest Company Slovakia
P.O. strain 6	CHINA BLACK	Mr. Rajtár, Mycoforest Company Slovakia
P.O. strain 7	PL-27	commercial strain
P.O. strain 8		isolate from the market, Slovakia
P.O. strain 9		origin unknown
P.O. strain 10	MEY 2191	Mr. Rajtár, Mycoforest Company Slovakia
P.O. strain 11	GIZA	Mr. Rajtár, Mycoforest Company Slovakia
P.O. strain 12	K12	Mr. Rajtár, Mycoforest Company Slovakia
P.O. strain 13	RH	Mr. Rajtár, Mycoforest Company Slovakia
P.O. strain 14	K6	Mr. Rajtár, Mycoforest Company Slovakia
P.O. strain 15		origin unknown
P.O. strain 16		origin unknown
P.O. strain 17		origin unknown
P.O. strain 18	P-84	Mr. Rajtár, Mycoforest Company Slovakia
P.O. strain 19		origin unknown
P.O. strain 20		origin unknown
P.O. strain 21		origin unknown, China 4
P.O. strain 22	PO-DD-1/1	Crop Research Institute, Czech Republic
P.O. strain 23	PO-SV-1/1	Crop Research Institute, Czech Republic
P.O. strain 24	PO-PH-1/1A	Crop Research Institute, Czech Republic
P.O. strain 25	PO-HOR-1/2	Crop Research Institute, Czech Republic
P.O. strain 26	PO-HOR-2/4	Crop Research Institute, Czech Republic
P.O. strain 27	PO-HD-1/1A	Crop Research Institute, Czech Republic
P.O. strain 28	PO-HD-2/1	Crop Research Institute, Czech Republic
P.O. strain 29	PO-MV-1/1A	Crop Research Institute, Czech Republic
P.O. strain 30	PO-SK-1	Crop Research Institute, Czech Republic
P.O. strain 31	PO-SK-3	Crop Research Institute, Czech Republic
P.O. strain 32	PO-SK-5	Crop Research Institute, Czech Republic
P.O. strain 33	PO-PSB	Crop Research Institute, Czech Republic
P.O. strain 34	Po-OH--JR1A	Crop Research Institute, Czech Republic
P.O. strain 35	PO ŠMA	Crop Research Institute, Czech Republic
P.O. strain 36		origin unknown
P.O. strain 37		from Hlíva Huť, Crop Research Institute, Czech Republic
P.O. strain 38	210-ENV	dr. Havránek, 2009, Olomouc, Czech Republic
P.O. strain 39	93-PLV	dr. Havranek 2008, Pohořany, Crop Research Institute, Czech Republic
P.O. strain 40	PLM pl	dr. Petrželová 2016, PR Doubrava (from Moravičany-Bradlec), Crop Research Institute, Czech Republic
P.O. strain 41	PLNZ sp1	dr. Petrželová, 2016, CHKO Litovelské Pomoraví (from Nové Zámky a Nový Dvůr), Czech Republic
P.O. strain 42	PLO sp	Dr. Egertová, Sochor 2015, Olomoučany, Czech Republic
P.O. strain 43	PLP pl	dr. Jurková, 2013, Pohořany, Czech Republic
P.O. strain 44		dr. Semerdžieva, 1993, Czech Republic
P.O. strain 45		dr. G. Ritter, 1956, Schierke, Harz mountains, Germany
P.O. strain 46		dr. E. Jones, 1966, England, Great Britain
P.O. strain 47		dr. W. Luthart, 1959, České Budějovice, Czech Republic
P.O. strain 48		dr. Luthart, 1960, České Budějovice, Czech Republic
P.O. strain 49		dr. Torev, 1965, Plovdiv, Bulgaria
P.O. strain 50		dr. Ginterová, 1973, Svatý Jur near Bratislava, Slovakia
P.O. strain 51		dr. Semerdžieva, 1983, Gaštanica near Nitra, Slovakia
P.O. strain 52		dr. Ohira 1975, Shuzenzi-cho, Pref. Shizuoka, Japan
P.O. strain 53		dr. Semerdžieva, 1987, Trutnov-okolí, Czech Republic
P.O. strain 54		dr. Semerdžieva, 1985, Czech Republic
P.O. strain 55		isolate from the market, Slovakia, 2019, SPOREA, origin Poland
P.O. strain 56		isolate from the market, Slovakia, 2019, origin Slovakia
P.O. strain 57		isolate from the market, Slovakia, 2019, České houby, origin Czech Republic
P.O. strain 58		isolate from the market, Slovakia, 2019, České houby, from ČR, origin Czech Republic
P.O. strain 59		isolate from the market, Slovakia, 2019, Agaricus Gombatermelo Kft, origin Czech Republic

Source: Author of the work.

**Table 2:** The basic validation characteristics of the method.

The Basic Validation Characteristics of the Method																	
	Al	Ba	Ca	Cd	Co	Cr	Cu	Fe	Hg	K	Mg	Mn	Na	Ni	Pb	Se	Zn
LOD_1_ (mg kg^−1^ DM)	0.0071	0.0033	0.068	0.00040	0.0018	0.011	0.0030	0.0011	0.00020	0.51	0.00028	0.00026	0.18	0.0017	0.0076	0.0028	0.0069
LOQ_1_ (mg kg^−1^ DM)	0.024	0.011	0.23	0.0013	0.0060	0.037	0.0098	0.0038	0.00060	1.7	0.00092	0.00086	0.60	0.0054	0.025	0.0084	0.023
RSD (%)	4.0	2.0	0.91	0.10	0.46	2.8	5.0	2.8	3.4	0.70	0.66	8.2	4.3	0.42	1.9	6.2	1.6
CL	quadratic	linear	quadratic	linear	linear	linear	linear	linear	linear	linear	quadratic	linear	linear	linear	linear	new racional	linear
Wavelengths	396.152 Axial	455.403 Radial	422.673 Radial	214.438 Axial	238.892 Axial	284.325 Radial	324.754 Axial	238.204 Axial	253.65	766.490 Radial	280.270 Radial	257.610 Axial	818.326 Axial	221.647 Axial	182.205 Axial	196.00	213.856 Radial

Note: CL—calibration line, RSD—relative standard deviation. Source: Author of the work.

**Table 3:** The content of selected substances in the stipe and in the cap (mg kg^−1^ DM).

Average Contents in 59 Strains of *Pleurotus ostreatus*										
	Al	Ba	Ca	Cu	Fe	K	Mg	Mn	Na	Zn
stipe	4.9 ± 4.5	4.8 ± 1.3	1200 ± 560	5.3 ± 2.4	40 ± 21	26,000 ± 5200	1500 ± 340	2.8 ± 1.3	590 ± 120	41 ± 15
cap	5.5 ± 4.6	4.00 ± 0.96	1200 ± 920	6.2 ± 1.5	56 ± 23	34,000 ± 4200	1800 ± 230	6.9 ± 1.8	600 ± 160	81 ± 25
average	5.2 ± 4.6	4.4 ± 1.1	1200 ± 740	5.8 ± 2.0	48 ± 22	30,000 ± 4700	1700 ± 290	4.9 ± 1.6	600 ± 140	61 ± 20

Notes: Mean ± standard deviation (SD) of each variable is reported in correspondence with each experimental treatment. Source: Author of the work.

**Table 4:** Minimum concentrations of metals in the stipes and the caps of the monitored oyster mushroom strains.

**STIPE**										
	**Al**	**Ba**	**Ca**	**Cu**	**Fe**	**K**	**Mg**	**Mn**	**Na**	**Zn**
Strain PO	PO44	PO5	PO3	PO29	PO45	PO31	PO20	PO36	PO2	PO45
mg kg^−1^ DM	1.9	3.0	310	1.8	12	16,000	880	0.39	62	11
**CAP**										
	**Al**	**Ba**	**Ca**	**Cu**	**Fe**	**K**	**Mg**	**Mn**	**Na**	**Zn**
Strain PO	PO32	PO17	PO1	PO36	PO45	PO58	PO17	PO45	PO1	PO45
mg kg^−1^ DM	1.7	2.6	200	3.0	22	24,000	1400	2.8	46	140

Notes: Numerical indication of the *Pleurotus ostreatus* strain in Chapter “Methods”. Source: Author of the work.

**Table 5:** Maximum concentrations of metals in the stipes and the caps of the observed oyster mushroom strains.

**STIPE**										
	**Al**	**Ba**	**Ca**	**Cu**	**Fe**	**K**	**Mg**	**Mn**	**Na**	**Zn**
Strain PO	PO1	PO39	PO39	PO38	PO39	PO45	PO51	PO58	PO26	PO31
mg kg^−1^ DM	22.0	9.7	3200	12.0	120	40,000	2400	4.8	800	83
**CAP**										
	**Al**	**Ba**	**Ca**	**Cu**	**Fe**	**K**	**Mg**	**Mn**	**Na**	**Zn**
Strain PO	PO43	PO26	PO26	PO39	PO2	PO21	PO21	PO34	PO26	PO1
mg kg^−1^ DM	17.0	6.9	4600	18.0	110	49,000	2400	11	920	920

Notes: Numerical indication of the *Pleurotus ostreatus* strain in Chapter “Methods”. Source: Author of the work.

**Table 6:** Average metals content (mg kg^−1^ DM) in samples of fortified fruiting bodies with selenium.

*Pleurotus ostreatus* strain 2-‘Kryos B’—The Content of Selected Metals mg kg^−1^ DM											
Variant	Zn	Co	Ni	Hg	Cu	Mn	Fe	Cr	Pb *	Cd *	Se
C	35.0 ± 3.3 a	0.61 ± 0.16 a	0.68 ± 0.32 a	0.041 ± 0.0041 a	7.50 ± 0.96 a	8.20 ± 0.91 a	49.0 ± 6.5 a	0.89 ± 0.52 a	1.60 ± 0.57 ab	0.23 ± 0.046 ab	0.11 ± 0.036 a
X	36.0 ± 2.7 a	0.91 ± 0.16 b	1.10 ± 0.32 b	0.047 ± 0.0047 b	7.40 ± 0.89 a	7.90 ± 0.40 ab	49.0 ± 6.6 a	0.75 ± 0.25 ab	1.90 ± 0.31 a	0.25 ± 0.048 a	0.32 ± 0.13 b
Y	38.0 ± 2.3 a	0.99 ± 0.17 b	1.60 ± 0.24 c	0.048 ± 0.0048 b	7.40 ± 0.72 a	7.70 ± 0.39 b	48.0 ± 2.9 a	0.62 ± 0.16 bc	1.40 ± 0.38 b	0.19 ± 0.063 ab	0.48 ± 0.094 c
Z	37.0 ± 2.8 a	1.20 ± 0.19 c	1.80 ± 0.24 d	0.048 ± 0.0048 b	7.20 ± 0.69 a	7.70 ± 0.30 b	48.0 ± 3.8 a	0.47 ± 0.17 c	0.97 ± 0.61 c	0.18 ± 0.089 b	0.81 ± 0.20 d

Notes: C—variant with 0 mg dm^−3^ Se, X—variant with 0.5 mg dm^−3^ Se, Y—variant with 1.0 mg dm^−3^ Se, Z—variant with 2.0 mg dm^−3^ Se. Mean ± standard deviation of each variable is reported in correspondence with each experimental treatment. Along each column, values followed by different letters are significantly different at *p* < 0.05 according to LSD test in ANOVA (Statgraphic XVII), Source: * Author of the work [23].

## References

[1] Villas-Bôas S.G., Esposito E., Mitchell D.A. (2002). Microbial Conversion of Lignocellulosic Residues for Production of Animal Feeds. Anim. Feed Sci. Technol..

[2] Alam N., Amin R., Khan A., Ara I., Shim M.J., Lee M.W., Lee U.Y., Lee T.S. (2009). Comparative Effects of Oyster Mushrooms on Lipid Profile, Liver and Kidney Function in Hypercholesterolemic Rats. Mycobiology.

[3] Cheung P.C.K. (2009). Mushrooms as Functional Foods.

[4] Khatun K., Mahtab H., Khanam P.A., Sayeed M.A., Khan K.A. (2007). Oyster Mushroom Reduced Blood Glucose and Cholesterol in Diabetic Subjects. Mymensingh Med. J. MMJ.

[5] Hu S.H., Liang Z.C., Chia Y.C., Lien J.L., Chen K.S., Lee M.Y., Wang J.C. (2006). Antihyperlipidemic and Antioxidant Effects of Extracts from *Pleurotus Citrinopileatus*. J. Agric. Food Chem..

[6] Nosál’ová V., Bobek P., Cerná S., Galbavý S., Stvrtina S. (2001). Effects of Pleuran (Beta-Glucan Isolated from *Pleurotus ostreatus*) on Experimental Colitis in Rats. Physiol. Res..

[7] Ajayi O.T., Ajayi O.T. (2021). Staphylococcus Aureus Propriétés Antibactériennes et Thérapeutiques In Vivo de P. Ostreatus Contre *Staphylococcus Aureus*. Res. J. Health Sci..

[8] Vlasenko V.A., Ilyicheva T.N., Zmitrovich I.v., Turmunkh D., Dondov B., Teplyakova T.v., Enkhtuya O., Nyamsuren K., Samiya J., Altangerel U. (2021). First Data on Antiviral Activity of Aqueous Extracts from Medicinal Mushrooms from the Altai Mountains in Russia against Influenza Virus Type A. Int. J. Med. Mushrooms.

[9] Joo Seo D., Choi C., Antiviral C., Baz M., Mifsud E. (2021). Antiviral Bioactive Compounds of Mushrooms and Their Antiviral Mechanisms: A Review. Viruses.

[10] Wang H., Gao J., Ng T.B. (2000). A New Lectin with Highly Potent Antihepatoma and Antisarcoma Activities from the Oyster Mushroom *Pleurotus Ostreatus*. Biochem. Biophys. Res. Commun..

[11] Mattila P., Salo-Väänänen P., Könkö K., Aro H., Jalava T. (2002). Basic Composition and Amino Acid Contents of Mushrooms Cultivated in Finland. J. Agric. Food Chem..

[12] Ahmed M.G., Yossef H.E., Ibrahim H.H. (2010). Protective Effects of Mushroom and Their Ethyl Extract on Aging Compared with L-Carnitine. Int. J. Nutr. Metab..

[13] Jayakumar T., Aloysius Thomas P., Geraldine P. (2007). Protective Effect of an Extract of the Oyster Mushroom, Pleurotus Ostreatus, on Antioxidants of Major Organs of Aged Rats. Exp. Gerontol..

[14] Audrey T.T.K., Calixte E.N.H., André-Ledoux N., Nico N.F., Paul M.F. (2021). Proximate and Minerals Composition of 12 Wild Mushrooms from the Noun Division, West Region in Cameroon Nutrient Content of Mushrooms from West Region, Cameroon Composition Proximale et Minérale de 12 Champignons Sauvages du Département du Noun, Région de l’Ouest-Cameroun. Cameroon J. Biol. Biochem. Sci..

[15] Mleczek M., Budka A., Kalač P., Siwulski M., Niedzielski P. (2020). Family and Species as Determinants Modulating Mineral Composition of Selected Wild-Growing Mushroom Species. Environ. Sci. Pollut. Res..

[16] Santos M.P.O., Santos M.V.N., Matos R.S., van der Maas A.S., Faria M.C.S., Batista B.L., Rodrigues J.L., Bomfeti C.A. (2021). Pleurotus Strains with Remediation Potential to Remove Toxic Metals from Doce River Contaminated by Samarco Dam Mine. Int. J. Environ. Sci. Technol..

[17] Krejsa J., Šíma J., Kobera M., Šeda M., Svoboda L. (2021). Detrimental and Essential Elements in Fruiting Bodies of Mushrooms with Ecological Relationship to Birch (*Betula* sp.) Collected in the Bohemian Forest, the Czech Republic. Environ. Sci. Pollut. Res..

[18] Demková L., Árvay J., Hauptvogl M., Michalková J., Šnirc M., Harangozo Ľ., Bobuľská L., Bajčan D., Kunca V. (2021). Mercury Content in Three Edible Wild-Growing Mushroom Species from Different Environmentally Loaded Areas in Slovakia: An Ecological and Human Health Risk Assessment. J. Fungi.

[19] Ralston N.V., Raymond L.J. (2010). Dietary Selenium’s Protective Effects against Methylmercury Toxicity. Toxicology.

[20] Hegedűsová A., Mezeyová I., Hegedűs O., Andrejiová A., Juríková T., Golian M., Lošák T. (2017). Increasing of Selenium Content and Qualitative Parameters in Garden Pea (*Pisum Sativum* L.) after Its Foliar Application. Acta Sci. Pol. Hortorum Cultus.

[21] Yan H., Chang H. (2012). Antioxidant and Antitumor Activities of Selenium- and Zinc-Enriched Oyster Mushroom in Mice. Biol. Trace Elem. Res..

[22] Falandysz J. (2008). Selenium in Edible Mushrooms. J. Environ. Sci. Health Part C.

[23] Golian M., Hegedűsová A., Trochcová M., Maťová A., Šlosár M. (2019). The Influence of Selenium on Selected Heavy Metals Cumulation in Oyster Mushroom Fruiting Bodies. Potravin. Slovak J. Food Sci..

[24] Mocak J., Bond A.M., Mitchell S., Scollary G., Bond A.M. (1997). A Statistical Overview of Standard (IUPAC and ACS) and New Procedures for Determining the Limits of Detection and Quantification: Application to Voltammetric and Stripping Techniques. Pure Appl. Chem..

[25] Hegedus O., Hegedusová A., Jakabová S., Vargová A., Pernyeszi T., Boros B. (2010). Evaluation of an HPIC Method for Determination of Nitrates in Vegetables. Chromatographia.

[26] Srikram A., Supapvanich S. (2016). Proximate Compositions and Bioactive Compounds of Edible Wild and Cultivated Mushrooms from Northeast Thailand. Agric. Nat. Resour..

[27] Singh A., Research P.D., Singh I.S., Pradesh U., Singh S. (2021). Nutritional and Health Importance of Fresh and Dehydrated Oyster Mushroom (*Pleurotus florida*). J. Curr. Res. Food Sci..

[28] Gogavekar S.S., Rokade S.A., Ranveer R.C., Ghosh J.S., Kalyani D.C., Sahoo A.K. (2014). Important Nutritional Constituents, Flavour Components, Antioxidant and Antibacterial Properties of Pleurotus Sajor-Caju. J. Food Sci. Technol..

[29] Yang S., Sun X., Shen Y., Chang C., Guo E., La G., Zhao Y., Li X. (2017). Tolerance and Removal Mechanisms of Heavy Metals by Fungus Pleurotus Ostreatus HAAS. Water Air Soil Pollut..

[30] Baldrian P., Gabriel J. (2002). Copper and Cadmium Increase Laccase Activity in *Pleurotus ostreatus*. FEMS Microbiol. Lett..

[31] Gucia M., Jarzyńska G., Kojta A.K., Falandysz J. (2012). Temporal Variability in 20 Chemical Elements Content of Parasol Mushroom (*Macrolepiota Procera*) Collected from Two Sites over a Few Years. J. Environ. Sci. Health Part B.

[32] Ahmad Zakil F., Xuan L.H., Zaman N., Alan N.I., Salahutheen N.A.A., Sueb M.S.M., Isha R. (2022). Growth Performance and Mineral Analysis of *Pleurotus ostreatus* from Various Agricultural Wastes Mixed with Rubber Tree Sawdust in Malaysia. Bioresour. Technol. Rep..

[33] Reguła J., Siwulski M. (2007). Dried shiitake (*Lentinulla edodes*) and oyster (*Pleurotus ostreatus*) mushrooms as a good source of nutrient. Acta Sci. Pol. Technol. Aliment..

[34] Arnold M., Rajagukguk Y.V., Gramza-Michałowska A. (2021). Functional Food for Elderly High in Antioxidant and Chicken Eggshell Calcium to Reduce the Risk of Osteoporosis—A Narrative Review. Foods.

[35] Patil S.S., Ahmed S.A., Telang S.M., Baig M.M.V. (2010). The Nutritional Value of *Pleurotus ostreatus* (Jacq.: Fr.) Kumm Cultivated on Different Lignocellulosic Agro-Wastes. Innov. Rom. Food Biotechnol..

[36] Riaz N., Guerinot M. (2021). lou All Together Now: Regulation of the Iron Deficiency Response. J. Exp. Bot..

[37] Raman J., Jang K.Y., Oh Y.L., Oh M., Im J.H., Lakshmanan H., Sabaratnam V. (2021). Cultivation and Nutritional Value of Prominent *Pleurotus* spp.: An Overview. Mycobiology.

[38] Budzyńska S., Siwulski M., Magdziak Z., Budka A., Gąsecka M., Kalač P., Rzymski P., Niedzielski P., Mleczek M. (2021). Influence of Iron Addition (Alone or with Calcium) to Elements Biofortification and Antioxidants in Pholiota Nameko. Plants.

[39] Sanglimsuwan S., Yoshida N., Morinaga T., Murooka Y. (1993). Resistance to and Uptake of Heavy Metals in Mushrooms. J. Ferment. Bioeng..

[40] Árvay J., Hauptvogl M., Šnirc M., Gažová M., Demková L., Bobuľská L., Hrstková M., Bajčan D., Harangozo Ľ., Bilčíková J. (2021). Determination of elements in wild edible mushrooms: Levels and risk assessment. Microbiol. Biotechnol. Food Sci..

[41] Wesołowska M., Filipczyk P., Zaguła G., Puchalski C., Dżugan M. (2021). Health safety of edible wild mushrooms collected from the industrial area. J. Microbiol. Biotechnol. Food Sci..

[42] Rózsa M., Măniuțiu D.-N., Egyed E. (2021). Influence of magnesium (Mg) source on the *Cordyceps militaris* (L.) mushroom mycelium growth. Curr. Trends Nat. Sci..

[43] Włodarczyk A., Krakowska A., Sułkowska-Ziaja K., Suchanek M., Zięba P., Opoka W., Muszyńska B. (2020). *Pleurotus* spp. Mycelia Enriched in Magnesium and Zinc Salts as a Potential Functional Food. Molecules.

[44] Keskin F., Sarikurkcu C., Akata I., Tepe B. (2021). Metal Concentrations of Wild Mushroom Species Collected from Belgrad Forest (Istanbul, Turkey) with Their Health Risk Assessments. Environ. Sci. Pollut. Res..

[45] Alaimo M.G., Dongarrà G., la Rosa A., Tamburo E., Vasquez G., Varrica D. (2018). Major and Trace Elements in Boletus Aereus and Clitopilus Prunulus Growing on Volcanic and Sedimentary Soils of Sicily (Italy). Ecotoxicol. Environ. Saf..

[46] Mleczek M., Budka A., Siwulski M., Mleczek P., Gąsecka M., Jasińska A., Kalač P., Sobieralski K., Niedzielski P., Proch J. (2020). Investigation of Differentiation of Metal Contents of Agaricus Bisporus, Lentinula Edodes and *Pleurotus ostreatus* Sold Commercially in Poland between 2009 and 2017. J. Food Compos. Anal..

[47] Ernst W.H.O., Verkleij J.A.C., Schat H. (1992). Metal Tolerance in Plants. Acta Bot. Neerl..

[48] Koštíř J. (1974). Biochemie.

[49] Baker A.J.M. Accumulators and Excluders-Strategies in the Response of Plants to Heavy Metals. https://www.scirp.org/(S(lz5mqp453edsnp55rrgjct55))/reference/ReferencesPapers.aspx?ReferenceID=1898610.

[50] Melgar M.J., Alonso J., García M.A. (2016). Cadmium in Edible Mushrooms from NW Spain: Bioconcentration Factors and Consumer Health Implications. Food Chem. Toxicol..

[51] Kalač P. (2013). A Review of Chemical Composition and Nutritional Value of Wild-Growing and Cultivated Mushrooms. J. Sci. Food Agric..

[52] Raiesi F., Sadeghi E. (2019). Interactive Effect of Salinity and Cadmium Toxicity on Soil Microbial Properties and Enzyme Activities. Ecotoxicol. Environ. Saf..

[53] Luo Y., Rimmer D.L. (1995). Zinc-Copper Interaction Affecting Plant Growth on a Metal-Contaminated Soil. Environ. Pollut..

[54] Manzi P., Aguzzi A., Pizzoferrato L. (2001). Nutritional Value of Mushrooms Widely Consumed in Italy. Food Chem..

[55] García M.A., Alonso J., Melgar M.J. (2015). Radiocaesium Activity Concentrations in Macrofungi from Galicia (NW Spain): Influence of Environmental and Genetic Factors. Ecotoxicol. Environ. Saf..

[56] Alonso J., García M.A., Pérez-López M., Melgar M.J. (2003). The Concentrations and Bioconcentration Factors of Copper and Zinc in Edible Mushrooms. Arch. Environ. Contam. Toxicol..

[57] Sarikurkcu C., Copur M., Yildiz D., Akata I. (2011). Metal Concentration of Wild Edible Mushrooms in Soguksu National Park in Turkey. Food Chem..

[58] Zhu F., Qu L., Fan W., Qiao M., Hao H., Wang X. (2011). Assessment of Heavy Metals in Some Wild Edible Mushrooms Collected from Yunnan Province, China. Environ. Monit. Assess..

[59] Pavlik M., Krupova D., Malucka L.U., Pavlik M., Rajtar M. Preliminary Mycoremediation Evaluation of Waste Ash from Thermal Power Plant Utilising the Oyster Mushroom. Proceedings of the Multidisciplinary Scientific GeoConference Surveying Geology and Mining Ecology Management, SGEM.

[60] Koutrotsios G., Danezis G., Georgiou C., Zervakis G.I. (2020). Elemental Content in Pleurotus Ostreatus and Cyclocybe Cylindracea Mushrooms: Correlations with Concentrations in Cultivation Substrates and Effects on the Production Process. Molecules.

[61] Rashid M.H., Rahman M.M., Correll R., Naidu R. (2018). Arsenic and Other Elemental Concentrations in Mushrooms from Bangladesh: Health Risks. Int. J. Environ. Res. Public Health.

